# Spatial–Spectral Feature Refinement for Hyperspectral Image Classification Based on Attention-Dense 3D-2D-CNN

**DOI:** 10.3390/s20185191

**Published:** 2020-09-11

**Authors:** Jin Zhang, Fengyuan Wei, Fan Feng, Chunyang Wang

**Affiliations:** School of Surveying and Land Information Engineering, Henan Polytechnic University, Jiaozuo 454003, China; 211804010001@home.hpu.edu.cn (J.Z.); weify@hpu.edu.cn (F.W.); 211804010008@home.hpu.edu.cn (F.F.)

**Keywords:** hyperspectral image classification, deep learning, 3D-2D-CNN, residual connection, attention mechanism, spatial–spectral feature refinement

## Abstract

Convolutional neural networks provide an ideal solution for hyperspectral image (HSI) classification. However, the classification effect is not satisfactory when limited training samples are available. Focused on “small sample” hyperspectral classification, we proposed a novel 3D-2D-convolutional neural network (CNN) model named AD-HybridSN (Attention-Dense-HybridSN). In our proposed model, a dense block was used to reuse shallow features and aimed at better exploiting hierarchical spatial–spectral features. Subsequent depth separable convolutional layers were used to discriminate the spatial information. Further refinement of spatial–spectral features was realized by the channel attention method and spatial attention method, which were performed behind every 3D convolutional layer and every 2D convolutional layer, respectively. Experiment results indicate that our proposed model can learn more discriminative spatial–spectral features using very few training data. In Indian Pines, Salinas and the University of Pavia, AD-HybridSN obtain 97.02%, 99.59% and 98.32% overall accuracy using only 5%, 1% and 1% labeled data for training, respectively, which are far better than all the contrast models.

## 1. Introduction

Recently, deep learning methods represented by convolutional neural networks (CNNs) have made a breakthrough in computer vision, showing great superiority in the image processing area [1,2,3]. Therefore, the research on the CNN models has attracted more and more attention, which also makes the application of CNN penetrate into various subareas of image processing, for example, remote sensing image processing area [4]. Hyperspectral image classification has always been one of the hotspots in the remote sensing community. At present, the CNN based hyperspectral classification methods are booming [5]. However, hyperspectral images suffer from a large number of spectral bands, large data size, high redundancy, high nonlinearity and the “small sample problem”, the pixel-wise classification of which is still challenging [6].

The convolutional neural network can automatically learn hierarchical abstract features from the raw image, which provides an ideal solution for feature extraction in computer vision. In 2012, a deep learning model named AlexNet [7] showed an excellent classification result in the ImageNet dataset, which is a huge collection of natural images. Since then, innovative networks have emerged in an endless stream, constantly inspiring the paradigm of feature extraction and reuse. In 2015, He et al. [8] proposed ResNet, solving the training problem of deep networks by introducing a residual connection. In ResNet, feature fusion is realized by pixelwise-addition of different feature maps. In 2017, Huang et al. [9] proposed DenseNet, which made it possible for feature reusing and provided another way for feature fusion, which is realized by the concatenation of different feature maps. In recent years, the above mentioned two feature fusion methods, which are proposed in ResNet and DenseNet, that have been widely used in the tasks of image classification [10,11], semantic segmentation [12,13], object detection [14,15], etc. Additionally, they are served as the standard patterns of feature extraction based on CNN. As milestones in the design of CNN models, the ideas behind ResNet and DenseNet are also radiating beyond the natural image processing area [16,17]. At present, the research of feature extraction and feature fusion for specific task or specific data is still a hot topic in the field of computer vision.

Hyperspectral image classification is the hotspot in remote sensing image interpretation and is of great difficulty. Its purpose is to assign an accurate label to each pixel in the image and then divide the image into areas with different ground object semantic identification [7]. Currently, the convolutional neural network has been successfully applied to the tasks of hyperspectral image classification [18,19,20,21]. In hyperspectral image (HSI) classification, the convolutional neural network acts as an “information distiller”, gradually extracting high-level abstract semantic features with the deepening of the network. In this process, the hyperspectral images with a huge amount of data are transformed, the irrelevant information is filtered out, and the useful information is enlarged and refined [22]. Prior to deep learning methods, traditional methods mostly used a linear discriminant analysis [23], such as the principal component analysis [24] and independent component analysis [25], to extract features. Additionally, they used a shallow classifier [26,27,28] to complete classification. These methods rely on manual designed features. For complex and diverse hyperspectral data, it is difficult to find a universal feature extraction method using such a route. Convolution neural network, which can learn features from HSI autonomously, provides a good solution for feature extraction. The HSI classification models based on 1D-CNN [29] or 2D-CNN [30] can achieve considerable classification results by automatically extracting features from hyperspectral images, but along with a degree of spatial or spectral information loss. In order to fully utilize spatial and spectral information in hyperspectral images simultaneously, the 3D-CNN, which is used to process video data before, is introduced to HSI classification. Compared with 2D-CNN, 3D-CNN has a relatively large computation burden, but can better learn spectral features within a hyperspectral image, which result in better classification performance. Since then, 3D-CNN is widely applied on HSI classification, based on which many improved models are proposed.

Chen et al. [18] constructed a 3D-CNN model composed of 3D convolutional layers and 3D pooling layers, improving classification performance by means of deep exploration into spatial–spectral features. Deeper networks enable deeper and more robust features and the network structure needs careful designing to pretend the greatly rising of the parameters amount. Lee et al. [19] made good use of residual connection in the spectral feature learning and built a deeper network (Res-2D-CNN) by which deeper and more abstract features could be extracted. Liu et al. [31] introduce residual connections to 3D-CNN and built Res-3D-CNN, which is aimed at enhancing spatial–spectral feature learning. Zhong et al. [20] focused on the raw hyperspectral data without dimensionality reduction and built SSRN (spectral–spatial residual network). They introduced residual connection into the whole network and separate deep feature learning procedure into independent spatial feature learning and spectral feature learning. More discriminative features were learned by SSRN and the separated feature learning pattern has a significant impact on subsequent hyperspectral classification research. Recently, dense connections have attracted more attention from hyperspectral researchers [32]. Dense connection reduces the network parameters through a small convolution kernel number, and realizes efficient feature reuse through feature map concatenation, both of which alleviates the problem of model overfitting. Wang et al. [21] introduced a dense block into SSRN using dense connections and built FD-SSC (Fast Dense Spectral–Spatial Convolution Network). With the help of a dense connection, FD-SSC further enhanced the feature propagation and reuse, making it possible that deeper hierarchical spatial–spectral features are extracted. Besides the rational use of different residual connections, structural innovation is also an important aspect of the network optimization of CNN models for hyperspectral classification. Swalpa K et al. [33] proposed a novel hyperspectral feature extraction pattern, HybridSN, based on the combination of the 3D-CNN and 2D-CNN. HybridSN takes hyperspectral data after a dimensionality reduction as the input and has a relatively small computation burden. It concatenates the feature maps extracted by three successive 3D convolutional layers in the spectral dimension and then used a 2D convolutional layer to enhance the spatial feature learning. HybridSN, which only has four convolutional layers, achieved extremely high classification accuracy, demonstrating the great potential of the 3D-2D-CNN model in hyperspectral classification. Based on the 3D-2D-CNN, Feng et al. [34] proposed R-HybridSN (Residual-HybridSN) by means of rational use of non-identity residual connections, enriching the feature learning paths and enhancing the flow of spectral information in the network. In particular, R-HybridSN was equipped with depth separable convolution layers instead of traditional 2D convolutional layers, which further made it perform better in the small sample hyperspectral classification. However, the shallow features in R-HybridSN are not reused, so that the network structure of R-HybridSN can be further optimized.

Hu et al. [35] proposed squeeze-and-excitation networks and introduced the attention mechanism to the image classification network, winning the champion of 2017 ImageNet Large Scale Visual Recognition Competition. Recently, the attention mechanism [36] has been applied to the construction of HSI classification models. The attention mechanism is a resource allocation scheme, through which limited computing resources will be used to process more important information. Therefore, the attention mechanism module can effectively enhance the expression ability of the model without excessively increasing complexity. Wang et al. [37] constructed a spatial–spectral squeeze-and-excitation (SSSE) module to automatically learn the weight of different spectral and different neighborhood pixels to emphasize the meaningful features and suppress unnecessary ones so that the classification accuracy is improved effectively. Li et al. [38] added an attention module (Squeeze-and-Excitation block) respectively after the dense connection module used for shallow and middle feature extraction to emphasize effective features in the spectral bands, and then feed it to further deep feature extraction. The attention mechanism in the HSI classification model is used for finding more discriminative feature patterns in spectral or spatial dimension. However, the specific use of the attention mechanism, such as the location and calculation methods, has no mature theory and still needs further exploring.

Hyperspectral image labeling is laborious and time-consuming, therefore, labeled samples are always limited in classification tasks. How to use as few labeled samples as possible to achieve better classification results has been a research hotspot for a long time. Feng et al. [34] conducted vast experiments using different amounts of training samples and found that the degradation of the CNN model is very common when the sample size decreased. The main strategies for small sample hyperspectral classification include generative adversarial networks [39,40], semi-supervised learning [41,42] and network optimization [33,34]. The residual connection is the core of network optimization, and the purpose of network optimization is to facilitate feature fusion and feature reusing. Compared with the simple pipelined network, the well-designed model, which is more like a directed acyclic graph of layers, usually has a better classification effect [34]. Song et al. [43] proposed a hybrid residual network (HDRN), in which the residual connection is used in and between residual blocks. The rational use of residual connection in the HDRN makes it better able to cope with hyperspectral classification with limited training samples. Network optimization can be combined with other methods. Liu et al. [44] proposed a deep few-shot learning method, which is focused on “small sample” hyperspectral classification. The Res-3D-CNN model is utilized to extract spatial–spectral features and to learn a metric space for each class of objects. Therefore, network optimization has important research significance and constructing models with a more reasonable structure seems to be an effective solution for the “small sample” hyperspectral classification.

Based on the above observations, in order to explore a better topological structure, inspired by R-HybridSN and the attention mechanism, we proposed a novel model named AD-HybridSN (Attention-Dense-HybridSN) for “small sample problem” from the perspective of network optimization. Based on 3D-2D-CNN and the densely connected module, AD-HybridSN realized a more efficient feature reuse and feature fusion. Moreover, the attention mechanism was introduced to the 3D convolution part and 2D convolution part respectively so that the model can utilize the spectral features and spatial features in a targeted refinement circumstance. With fewer parameters, AD-HybridSN achieves better classification performance in the Indian Pines, Salinas and University of Pavia datasets.

## 2. Methodology

### 2.1. AD-HybridSN Model

**Figure 1:**
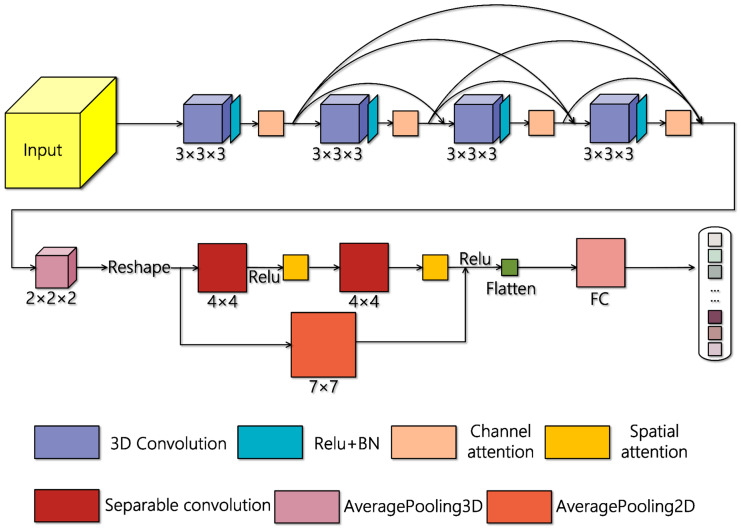
Illustration of the proposed Attention-Dense-HybridSN (AD-HybridSN).

Hyperspectral image classification is to assign a specific label to every pixel in hyperspectral images. The convolutional neural networks based hyperspectral classification models take small image patches as the input. Every hyperspectral image patch was composed of the spectral vectors within a predefined range and its land-use type was determined by its center pixel. The hyperspectral patch can be denoted as $P_{L\times W\times S}$, where L×W represents the spatial dimension and S represents the number of spectral bands. In our proposed model, the input data was processed by principal components analysis (PCA) in the spectral dimension, which greatly reduced the redundancy within hyperspectral data. The number of spectral bands decreased from S to C, and a different value of C has a great effect on the computational complexity. For the sake of equilibrium, we set L and W as 15 and C as 16 in our proposed network. Figure 1 shows the network structure of the proposed AD-HybridSN. AD-HybridSN is based on the 3D-2D-CNN feature extraction pattern and is composed of 6 convolutional layers. A 3D-Dense block composed of 4 3D convolutional layers was used for learning hierarchical spatial–spectral features. We introduced the channel attention method after every 3D convolutional layer to refine the extracted spatial–spectral features. Two subsequent depth separable convolutional layers supported by the spatial attention method enhanced the spatial information in the feature maps. Multiple residual connections were used in AD-HybridSN, which realized the feature reuse and spectral information compensation.

### 2.2. Convolutional Layers Used in Our Proposed Model

In the CNN based hyperspectral classification networks, the input hyperspectral image patch contains abundant spatial–spectral information. As for feature extraction from the hyperspectral image patch, different types of convolution have different characteristics. In 2D-CNN, the 2D convolution kernels were used to carry out convolution operation in the spatial dimension of the feature maps, and then the corresponding operation results of different channels were added by the pixel to obtain a 3D tensor. Assuming that the input data dimension was Z×Z×C, the 2D convolution kernel size was M×M, the kernel number was N and padding was not used, then the 3D tensor of which its size was (Z−M+1)×(Z−M+1)×N  was obtained. The value of the (x,y) position on the jth feature map at the ith layer,$\;value_{i,j}^{x,y}$, was calculated by Formula (1),

(1) $$value_{i,j}^{x,y}=\varphi \left(b_{i,j}+\sum_{\mu }\sum_{w=0}^{W_{i}-1}\sum_{l=0}^{L_{i}-1}\theta_{i,j,\mu }^{w,l}value_{\left(i-1\right),\mu }^{\left(x+w\right),\left(y+l\right)}\right)$$

where φ() represents the nonlinear activation function and $W_{i}$, $L_{i}$ represents the width and height of the convolutional kernel; $\theta_{i,j,\mu }^{w,l}$ represents the weight parameter at the position (l,w) on the jth feature map in the ith layer; $value_{\left(i-1\right),\mu }^{\left(x+w\right),\left(y+l\right)}$ represents the value at the (x+w,y+l) position in the μth feature map of the previous layer and $b_{i,j}$ represents the bias.

In the 3D-CNN based models, the convolution operation using the 3D convolution kernel not only acts on the spatial dimension, but also obtains the correlation information between several spectral bands. Suppose the dimension of the input hyperspectral image patch is Z×Z×C, and N 3D convolution kernels of which the kernel size is M×N×D was used for convolution operation. If padding is not used, then the output feature map is a 4D tensor and its dimension is (Z−M+1)×(Z−M+1)×(C−D+1)×D, the number of bands across the spectral dimension is fixed as D and the final result is a 4D tensor with a size of (Z−M+1)×(Z−M+1)×(C−D+1)×N. It is a remarkable fact that the number of bands crossed in spectral feature learning is fixed as D, which may be the limitation of the 3D convolutional kernel. Compared with the feature maps generated by 2D convolutional kernels, 3D convolutional kernel can better utilize spectral information. Therefore, the 3D-CNN based methods are more suitable for the classification of hyperspectral images with rich spectral information. In the 3D convolutional computation, the activation value at the (x,y,z) position on the jth feature map in the ith layer can be is calculated by Formula (2).

(2) $$value_{i,j}^{x,y,z}=\varphi \left(b_{i,j}+\sum_{\mu }\sum_{w=0}^{W_{i}-1}\sum_{l=0}^{L_{i}-1}\theta_{i,j,\mu }^{w,l,d}value_{\left(i-1\right),\mu }^{\left(x+w\right),\left(y+l\right),\left(z+d\right)}\right)$$

3D-CNN can make good use of the rich spectral information in hyperspectral data while extracting spatial information. However, the 3D convolution kernel has an extra spectral dimension compared with the 2D convolution kernel, which inevitably introduces more parameters and greatly increases the computational complexity [27]. Inspired by HybridSN, in consideration of making better use of the advantages of 3D-CNN and 2D-CNN respectively in feature extraction, we used the 3D-2D-CNN as the feature extraction pattern. Inspired by R-HybridSN, we used depth separable convolutional layers in AD-HybridSN. The depth separable convolution can be divided into depthwise convolution and pointwise convolution. Compared with traditional 2D convolutional layers, the depth separable convolutional layers have fewer parameters and less computational burden, which make it more suitable for hyperspectral data processing [34].

### 2.3. Multiscale Feature Reuse Module

The residual connection used in ResNet realized feature fusion through pixel-wise addition of feature maps at different layers, making it relatively easy to train deep networks. Based on the necessity of feature fusion, in the DenseNet proposed by Gao Huang et al., the correlations between feature maps are extended to some limitation by concatenating the outputs of any two layers in one dense block. On account of the feature fusion method of concatenating feature maps in the channel dimension, the features extracted by the earlier convolutional layers are completely preserved, which makes it possible for complete feature reuse. For a CNN model with t convolutional layers, the tth layer will receive all characteristic information from the 0 layer to the t−1 layer, as shown in Formula (3),

(3) $$X_{t}=H_{t}\left(\left[X_{0},X_{1},\ldots \ldots ,X_{t-1}\right]\right)$$

where $H_{t}()$ represents the computation of the tth layer and $\left[X_{0},X_{1},\ldots \ldots ,X_{t-1}\right]$ represents the concatenation result of all the previous layers in one dense block. This feature fusion method effectively enhances the free flow of information in the model. However, current DenseNet based models adopt local dense connections, which means dense connections only exist within each dense block. This is inevitable because globally dense connections consume a lot of memory and have a severe feature redundancy problem.

From the perspective of the receptive field of the feature maps, in AD-HybridSN, we used four 3D convolutional layers in one dense block and each layer included 12 convolutional kernels, with a size of 3×3×3. With the convolution operation layer by layer, the spatial and spectral receptive fields of each set of feature maps aggressively increase. For example, after the first convolution operation, a set of feature maps, of which the receptive field was 3×3×3, were obtained. If there is no dense connection, the receptive field of the subsequent output feature maps will be 5×5×5, 7×7×7 and 9×9×9. In fact, due to the reuse of shallow features, the actual receptive field will be smaller. At the same time, multiscale features were sufficiently reused through dense concatenation operation in the dense block.

### 2.4. Attention Mechanism

Currently, the attention mechanism has been successfully applied to the area of computer vision based on convolutional neural networks. The attention mechanism can be used to readjust feature maps generated by some layers of a neural network, which make it able to detect specific channel or spatial features [45,46]. The attention mechanism can be roughly divided into spatial attention and channel attention [34]. In our proposed model, channel attention was introduced to the refactor and refines the spatial–spectral features extract by every convolutional layer in the dense block and spatial attention was utilized to discriminate spatial information within the features generated by the depth separable convolutional layers.

#### 2.4.1. Channel Attention Module

As mentioned above, the feature map extracted by a single 3D convolution kernel is modeled as a 3D cube, which can learn detailed features and correlation information across spectral bands of hyperspectral data to some extent. Take the 3×3×3 convolutional kernel as an example, the same parameters are used for single channel 3D hyperspectral data during which each convolution operation covers three spectral bands. As the band span of the spectral features characterized by a single convolutional layer is fixed, the spectral feature mining has been limited to some extent. Therefore, in order to further refine the extracted spatial–spectral features, feature maps of all channels were concatenated in the spectral dimension to form a 3D tensor. The reshaped 3D tensor had a large channel number, which was equal to the original channel number times the original spectral band number. Then channel attention was introduced to assign a specific weight for each channel.

**Figure 2:**
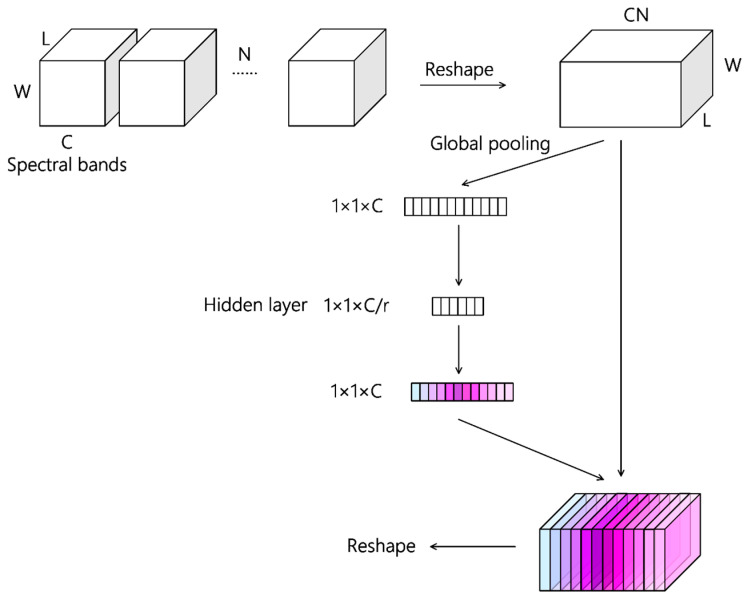
The overall architecture of the channel attention mechanism used in AD-HybridSN. The dimension of the input feature map is L×W×C×N (batchsize is not shown) and after the reshaping operation, the dimension of the new feature map will be L×W×(CN). After global pooling operation and two fully connected layers, specific weights are generated for every channel and the weight vector will be multiplied with the reshaped 3D tensor to complete feature refinement. After feature refinement, the 3D tensor will be reshaped to a 4D tensor, of which the dimension is L×W×C×N.

Figure 2 is a schematic diagram of the channel attention mechanism used in this article. Let the dimension of the feature map generated by 3D convolutional layers be B×L×W×C×N, where B represents batchsize, L×W represents the spatial dimension of the feature map, C represents the spectral dimension and N represents the number of convolution kernels. In our proposed method, the 5D feature map will be reshaped to be a 4D tensor and its dimension will be B×L×W×(CN), where CN is the new channel number. Then channel attention is applied to the new 4D tensor to generate a specific weight for each channel. Firstly, global average pooling was performed on the 4D tensor to obtain channel-wise representation. Then, after two fully connected layers, the latter of which used sigmoid as the activation function, the channel-wise representation was converted to the weights of different channel. Finally, the reshaped input feature map was multiplied by the obtained weight vector to complete the refactoring of spatial–spectral features. Take the settings of the proposed model as an example, the dimension of the feature map generated by the first convolutional layer was 15×15×16×12. Twelve weights will be produced if the channel attention mechanism was directly used but the number of weights increased to 192 after reshaping. Therefore, the feature map reshaping was helpful to refine the spectral feature within the original feature maps.

#### 2.4.2. Spatial Attention Module

**Figure 3:**
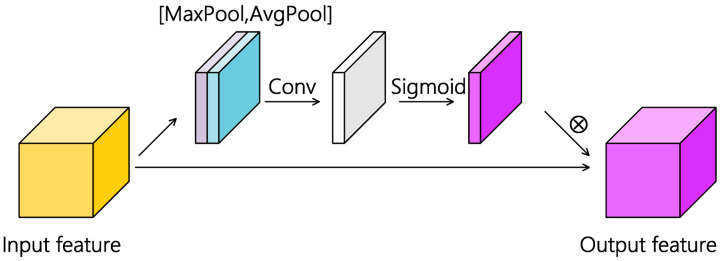
The overall architecture of the spatial mechanism used in AD-HybridSN. The dimension of the input feature map is L×W×C (batchsize is not shown). After max pooling, average pooling and concatenation operation, the dimension of the new feature map will be 1×1×2C. A convolutional layer that has only one kernel with a sigmoid activation function is used to learn where more attention is needed. The obtained weights are multiplied with the input feature map to complete spatial feature refinement.

Figure 3 shows a schematic of the spatial attention module. Suppose F represents the reshaped feature maps generated by the first depth separable convolutional layer, of which the dimension can be denoted as B×L×W×C. The processing process of the attention mechanism in this paper can be summarized as Formula (4),

(4) $$F^{\prime }=SA\left(F\right)⊗F$$

where SA() represents spatial attention, ⊗ represents the matrix multiplication operation and $F^{\prime }$ represents the refined feature maps. Take the first depth separable convolutional layer as an example, global average pooling and global max pooling were performed after the convolutional operation and the feature map dimension became B×1×1×C for each pooling operation. Then the two pooling results were concatenated to build an efficient feature descriptor, namely the spatial attention feature map. The spatial attention feature map will be further processed by a single convolutional kernel so that the location that needs strengthening or suppressing can be highlighted. The above computational process can be summarized as Formula (5),

(5) $$SA\left(F\right)=sigmoid\left(f^{4\times 4}\left(\left[AvgPool\left(F\right);MaxPool\left(F\right)\right]\right)\right)$$

where [;] represents the concatenation operation of two feature maps, Avgpool () and MaxPool () represent the corresponding pooling operation, $f^{4\times 4}$ represents the convolutional operation and sigmoid () is the activation function.

In our proposed model, the feature maps extracted by 3D convolutional layers contain abundant spectral information. During the subsequent 2D operation, spectral information suffers some loss while spatial information is strengthened, so residual connection was used to compensate spectral information, which in fact constructed a dual-path feature learning pattern. However, this simple dual-path feature extraction pattern was not able to learn the refined spatial feature. The convolutional kernels in the depth separable convolutional layers could not cover the whole feature map, so we used the spatial attention mechanism to refine the feature map point by point.

## 3. Datasets and Contrast Models

**Figure 4:**
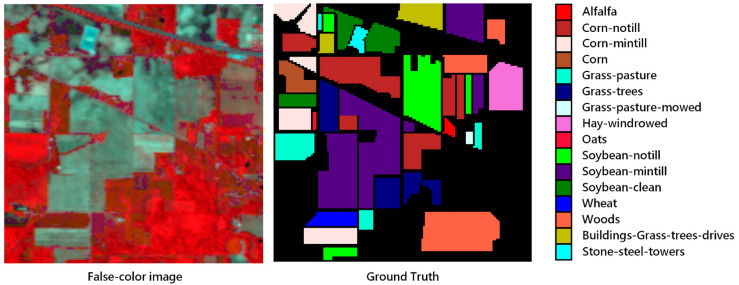
False-color image and color coding for Indian Pines.

**Figure 5:**
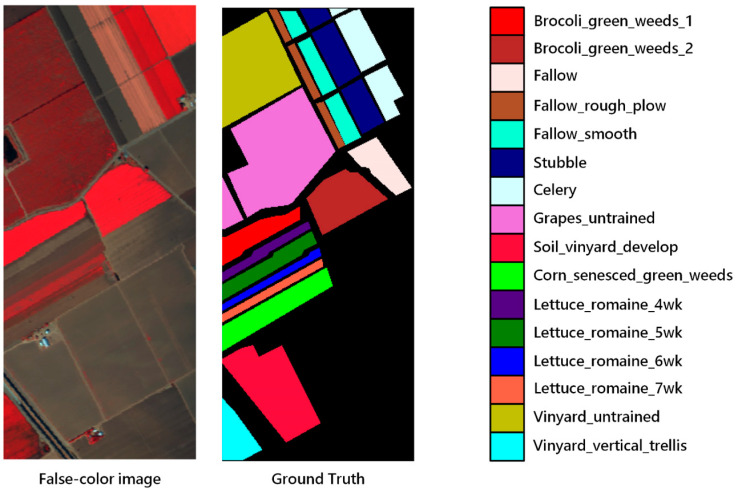
False-color image and color coding for Salinas.

**Figure 6:**
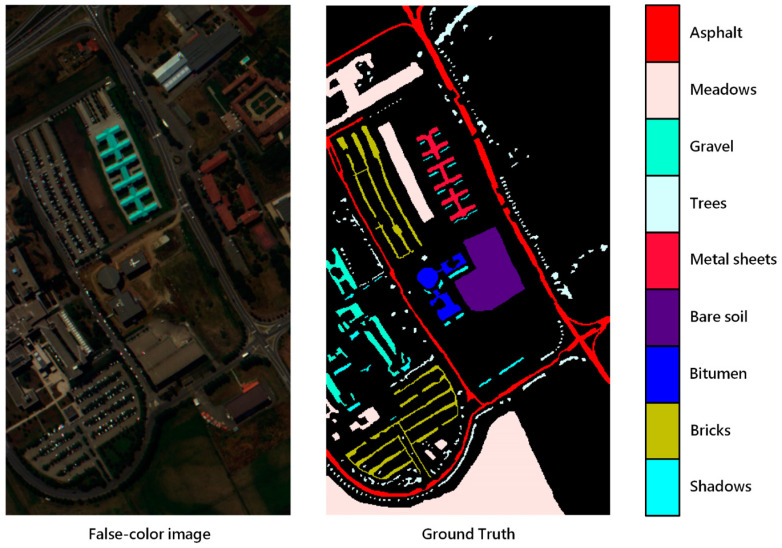
False-color image and color coding for the University of Pavia.

To observe the performance of the proposed model, three datasets: Indian Pines, Salinas and the University of Pavia were used in our experiment. Indian Pines was collected by the AVIRIS sensor in Indiana. The image had 145 pixels × 145 pixels and contained 224 bands. Apart from 24 bands absorbed by water vapor, 200 bands were available for classification. The spatial resolution of the image was 20 m, and the spectral coverage was 0.4–2.5 μm. The number of labeled samples was 10,249, which were divided into 16 categories, including crops and natural vegetation such as soybean, corn, wheat, alfalfa and pasture. The Salinas dataset was acquired in California by the AVIRIS sensor and contained 204 bands for classification without water vapor absorption bands. The image size was 512 × 127 and the spatial resolution was 3.7 m. It contained 16 types of ground objects in total, and the spectral coverage was the same as Indian Pines. The dataset mainly included vegetables, bare earth and vineyards. The University of Pavia dataset contained nine classes, with a total sample size of 42,776. It contained 610 pixels × 340 pixels, with a spatial resolution of 1.3 m and a spectral coverage range of 0.43–0.86 μm. The dataset was collected in urban areas, mainly including trees, asphalt roads, bricks, pastures, etc. Figure 4, Figure 5 and Figure 6 show the distribution of each ground objects on the Indian Pines, Salinas and University of Pavia.

In our experiments, for each image we divided all the pixels into three parts: training set, test set and validation set. The proportion of the training set and validation set of the Indian Pines, Salinas and University of Pavia datasets was 5%, 1% and 1% respectively and the remaining pixels served as a test set. The sample distribution of the three datasets for each class of ground object is shown in Table 1, Table 2 and Table 3. We used the HybridSN [32], R-HybridSN [33], Res-3D-CNN [31] and Res-2D-CNN [19] as contrast models to verify the classification performance of AD-HybridSN.

## 4. Experimental Results and Discussion

In our experiment, training sets of HybridSN, R-HybridSN, Res-3D-CNN and Res-2D-CNN, such as window size, training epoch, etc., were consistent with the corresponding papers. In addition, we used the Adam optimizer and the learning rate was set to 0.001. In order to observe the performance of our model, we trained 100 epochs and used ReLU as an activation function in AD-HybridSN. In all experiments, we monitored the validation accuracy and saved the model with the highest verification accuracy.

### 4.1. Experimental Results

Three indexes were used to measure the accuracy of models, namely, overall accuracy (OA), average accuracy (AA) and Kappa coefficient (Kappa). OA represents the proportion of the number of samples that were correctly classified by the model. AA stands for the average precision of all land objects. KAPPA is an accuracy measure based on the confusion-matrix, which represents the percentage of errors reduced by classification versus a completely random classification.

**Figure 7:**
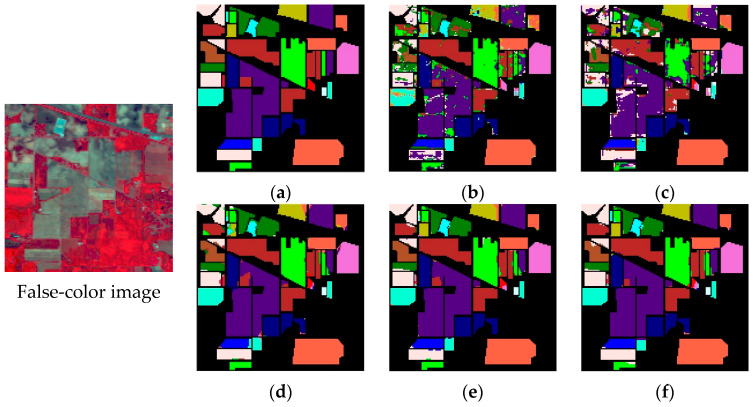
The classification maps of Indian Pines. (**a**) Ground truth. (**b**–**f**) Predicted classification maps for Res-2D-CNN, Res-3D-CNN, HybridSN, R-HybridSN and AD-HybridSN respectively.

**Figure 8:**
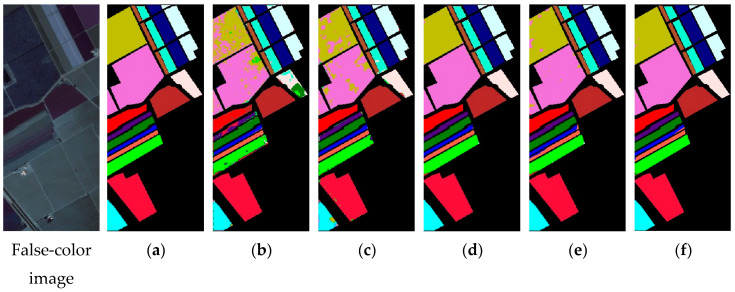
The classification maps of Salinas. (**a**) Ground truth. (**b**–**f**) Predicted classification maps for Res-2D-CNN, Res-3D-CNN, HybridSN, R-HybridSN and AD-HybridSN respectively.

**Figure 9:**
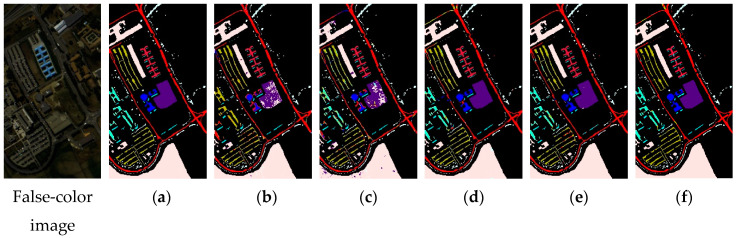
The classification maps of the University of Pavia. (**a**) Ground truth. (**b**–**f**) Predicted classification maps for Res-2D-CNN, Res-3D-CNN, HybridSN, R-HybridSN and AD-HybridSN respectively.

In order to avoid fluctuations caused by accidental factors as far as possible, we conducted 20 consecutive experiments. Table 4, Table 5 and Table 6 show the average indices and standard deviation of each model on three datasets. Figure 7, Figure 8 and Figure 9 show the false-color map, the ground truths and the classification results of each model for three datasets. We can tell by the data and predicted maps that the classification result of AD-HybridSN was more detailed and accurate in Indian Pines, Salinas and University of Pavia. Among the contrast models, the OA of Res-2D-CNN on the three datasets were lower than the other contrast models, indicating that the 2D-CNN model was not suitable for small sample hyperspectral classification. Secondly, the classification result of Res-3D-CNN was higher than that of Res-2D-CNN, indicating that the 3D-CNN model could explore spatial–spectral features of training samples more effectively. R-HybridSN was superior to the HybridSN in Indian Pines and University of Pavia, and the two models had a higher classification accuracy than Res-3D-CNN, to a certain extent, it proved that, compared with the model that used the 3D convolution kernel or 2D convolution kernel alone, the 3D-2D-CNN model was more suitable for the classification under the condition of small samples, and the reasonable use of the residual connection could effectively improve the classification performance of the 3D-2D-CNN model. In particular, the classification accuracy of R-HybridSN in Salinas was slightly lower than HybridSN and our proposed model AD-HybridSN effectively solved this problem. Among the three 3D-2D-CNN models, our proposed AD-HybridSN achieved the highest classification accuracies in three datasets. For example, the OA of AD-HybridSN was 0.26% and 2.71% higher than R-HybridSN and HybridSN in Indian Pines.

We further compared the experiment results of the three 3D-2D-CNN based models and drew the following conclusions. Firstly, unlike R-HybridSN, which had inferior classification accuracy than HybridSN on Salinas, the classification accuracy of AD-HybridSN was relatively balanced on three datasets. It further demonstrated the strong feature extraction ability of the dense block and the necessity of feature refinement module. What is more, AD-HybridSN had an uneven classification accuracy on different datasets. Using a similar amount of training samples in three datasets, the classification effect of Salinas was far better than Indian Pines. Thus, the generalization ability of AD-HybridSN needs to be further analyzed. Thirdly, compared with the other two 3D-2D-CNN models, AD-HybridSN had a tremendous improvement in small sample classes, such as the Stone-steel Towers in Indian Pines and Shadows in the University of Pavia. However, the classification accuracy of AD-HybridSN on some ground objects, such as oats and alfalfa in Indian Pines and Lettuce_romaine_7wk in Salinas, which was over that of R-HybridSN, was still lower than HybridSN, which needs to be further studied.

### 4.2. Discussion

It is proved that the classification performance of AD-HybridSN was superior to R-HybridSN, HybridSN and other contrast models through vigorous experiments. Therefore, the network structure of AD-HybridSN was conducive to improving classification accuracy, which needs to be further discussed. From the perspective of the network structure, the HybridSN is a 3D-2D-CNN model with a relatively concise structure, which contains only four convolutional layers; R-HybridSN has a relatively deeper and more complex structure, which is based on the non-identity residual connection and depth separable convolutional layers. It can be speculated from the experimental results that R-HybridSN had a better spatial–spectral feature learning ability. At the same time, the features extracted from the shallow network layers were not fully utilized, which may be the reason that the accuracy of R-HybridSN in the Salinas dataset was slightly lower than that of HybirdSN. AD-HybridSN is the redevelopment of R-HybridSN, based on which the dense block and attention module are introduced for feature reusing and refinement. As AD-HybridSN only has six convolutional layers, the structural advantage of our proposed network was verified. However, the effectiveness of attention module needs to be further verified.

In order to further verify the effectiveness of the attention module in our proposed model, we built a D-HybridSN to conduct model ablation experiments. In order to control the experimental variables, the only difference between D-HybridSN and AD-HybridSN was that the former had no attention module. Table 7 shows the accuracy of AD-HybridSN, D-HybridSN and R-HybridSN in three datasets and the proportion of the training sample used in this experiment was also 5%, 1% and 1% respectively. The classification accuracies of D-HybridSN were −0.42%, 0.66% and 0.27% higher than that of R-HybridSN in Indian Pines, Salinas and the University of Pavia respectively. From the comprehensive performance of models on the three datasets, the features extracted by D-HybridSN were more discriminative. Thus, it is further proved that, by means of reusing the spatial–spectral features in the network, the features from shallow layers were better utilized to contribute to classification. What is more, our proposed AD-HybridSN outperformed D-HybridSN in three datasets by 0.68%, 0.19% and 0.41% respectively, which indicate that the spatial–spectral features were further refined by the attention module that followed every convolutional layer.

Although AD-HybridSN has satisfactory overall accuracies on the three datasets, the classification of some ground objects was still unsatisfactory. This phenomenon may be attributed to the fixed network structure for different datasets, which may limit the targeted feature learning for different datasets with different spatial resolution and spectral conditions. Therefore, in the following research, the model integration method will be used to integrate the advantages of different networks, so as to comprehensively improve the classification accuracy of various ground objects. Besides, the fixed network structure might mean a fixed input size, which includes window sizes and a number of bands. That may further affect the ability of the model on learning spatial–spectral features from different datasets. Thus, how to learn features in a more flexible way needs to be further investigated in the aspect of network structure and hyperspectral image preprocessing.

In order to further verify the performance of AD-HybridSN under the “small-sample” condition, we further reduced the amount of training samples and conducted supplementary experiments. In Section 4.1 we showed the experiment results under unbalanced training sample cases, and we will further reduce the amount of training samples. Meanwhile, we will use balanced training samples, which means the amount of each ground objects are equal, to perform supplementary experiments. Due to that, 5% is the minimum proportion of Indian Pines to ensure that all ground objects have at least one sample and the classification accuracy of the University of Pavia is relatively low, we only used the University of Pavia in our supplementary experiments. In the unbalanced training sample experiment, the amount of labeled data decreased from 0.8% to 0.4%. In the balanced training sample experiment, we used 50, 40, 30 and 20 labeled data of each ground object respectively. Table 8 and Table 9 show the experiment results.

After analyzing the experimental results, we had the following findings:

(1) In our experiments, the classification accuracy of AD-HybridSN was the highest in both the unbalanced training sample case and balanced training sample case. Additionally, the classification accuracy of R-HybridSN was higher that of HybridSN, which was consistent with the experiment results of the University of Pavia in Section 4.1.

However, the classification accuracy of the three models showed great differences in the two kinds of experiments. By comparing the OA and AA value in the experiment result, we found that, in the balanced training sample case, the AA value was relatively higher, which was different from the experiment on the unbalanced training sample case. This phenomenon indicates that the sample distribution had a great influence on the classification results.

(2) The experiment results further indicate that the sample distribution was a valuable research issue in “small sample” hyperspectral classification. For now, we randomly split the hyperspectral data to obtain the training set, validation set and testing set. However, there was an ill-posed problem in the hyperspectral image. On the one hand, the amount of samples were unbalanced, on the other hand, the quality of samples were also unbalanced. Thus, selecting the best training sample combination from the labeled samples may alleviate the problem of the hyperspectral image ill-posed problem to a certain extent.

(3) We can tell by the experimental results that when the number of training samples was reduced to a certain extent, the classification accuracy of all models decreased in a cliff-like manner. Therefore, there is a limit to improve the classification accuracy of small samples only by network optimization. When the training samples were reduced to a certain extent, there were a large number of unlabeled samples that were not used. Thus, in the following research, we should focus on mining the information of unlabeled samples by combining semisupervised learning or an active learning strategy.

## 5. Conclusions

In this paper, in order to realize the efficient extraction and refinement of the spatial–spectral feature in the “small sample” hyperspectral image classification, we proposed an AD-HybridSN model from the perspective of network optimization. Based on the 3D-2D-CNN model, multifeature reuse was realized by a dense block. Besides, the 3D convolution and 2D convolution were respectively equipped with channel attention and spatial attention modules, thus the spatial–spectral features were further refined. We conducted a series of experiments on three open datasets: Indian Pines, Salinas and the University of Pavia. The experiment results show that the AD-HybridSN model had a better classification effect than all the contrast models. In Section 4.2, we further performed the supplementary experiment on a balanced training sample case. AD-HybridSN still had the best classification results when the amount of training samples decreased. However, the accuracy improvement brought by network optimization was limited, so other strategies should be combined to further improve the classification accuracy.

In our proposed model, the attention module was of great help to improve the accuracy of the hyperspectral classification under the “small sample” condition. However, in AD-HybridSN, only a simple attention module was used. In the future, we will further study the attention mechanism and a more targeted attention module could be designed and applied in the hyperspectral image classification experiment. In addition, the AD-HybridSN still has room for improvement in the classification of specific ground objects. In subsequent studies, we will combine semisupervised learning or active learning strategy to break through the bottleneck of network optimization. Moreover, the dense block and attention module are only preliminarily explored in AD-HybridSN. Network optimization is an open field with rapid development, we hope that the idea of AD-HybridSN can be further expanded.

## Figures and Tables

**Table 1:** Training, validation and testing sample numbers in Indian Pines.

Number	Name	Training	Validation	Testing	Total
1	Alfalfa	2	3	41	46
2	Corn-notill	71	72	1285	1428
3	Corn-min	42	41	747	830
4	Corn	12	12	213	237
5	Grass/Pasture	24	24	435	483
6	Grass/Trees	36	37	657	730
7	Grass/Pasture-mowed	2	1	25	28
8	Hay-windrowed	24	24	430	478
9	Oats	1	1	18	20
10	Soybean-notill	48	49	875	972
11	Soybean-mintill	123	122	2210	2455
12	Soybean-clean	30	29	534	593
13	Wheat	10	10	185	205
14	Woods	63	63	1139	1265
15	Building-Grass-Trees-Drives	19	20	347	386
16	Stone-steel Towers	5	4	84	93
**Total**		512	512	9225	10,249

**Table 2:** Training, validation and testing sample numbers in Salinas.

Number	Name	Training	Validation	Testing	Total
1	Brocoli_green_weeds_1	20	20	1969	2009
2	Brocoli_green_weeds_2	37	37	3652	3726
3	Fallow	20	20	1936	1976
4	Fallow_rough_plow	14	14	1366	1394
5	Fallow_smooth	27	27	2624	2678
6	Stubble	39	40	3880	3959
7	Celery	36	36	3507	3579
8	Grapes_untrained	113	112	11,046	11,271
9	Soil_vinyard_develop	62	62	6079	6203
10	Corn_senesced_green_weeds	33	33	3212	3278
11	Lettuce_romaine_4wk	11	10	1047	1068
12	Lettuce_romaine_5wk	19	20	1888	1927
13	Lettuce_romaine_6wk	9	9	898	916
14	Lettuce_romaine_7wk	11	10	1049	1070
15	Vinyard_untrained	72	73	7123	7268
16	Vinyard_vertical_trellis	18	18	1771	1807
**Total**		541	541	53,047	54,129

**Table 3:** Training, validation and testing sample numbers in University of Pavia.

Number	Name	Training	Validation	Testing	Total
1	Asphalt	66	66	6499	6631
2	Meadows	186	186	18,277	18,649
3	Gravel	21	21	2057	2099
4	Trees	30	31	3003	3064
5	Metal sheets	14	13	1318	1345
6	Bare soil	50	50	4929	5029
7	Bitumen	14	13	1303	1330
8	Bricks	37	37	3608	3682
9	Shadows	9	10	928	947
**Total**		427	427	41,922	42,776

**Table 4:** Classification results of different models in Indian Pines.

No.	Training Samples	Res-2D-CNN	Res-3D-CNN	HybridSN	R-HybridSN	AD-HybridSN
1	2	12.07	27.07	61.10	45.73	49.02
2	71	78.46	83.45	92.20	95.44	94.79
3	42	60.00	75.37	96.48	97.41	98.21
4	12	42.84	56.06	77.11	93.17	96.46
5	24	81.87	92.90	94.30	96.71	96.94
6	36	92.30	96.50	97.27	99.30	98.20
7	2	27.40	67.80	89.00	98.60	100.00
8	24	99.44	98.27	97.97	100.00	99.90
9	1	3.61	60.28	83.89	64.44	65.28
10	48	74.42	83.22	95.18	96.01	95.57
11	123	82.74	89.38	97.78	98.31	99.03
12	30	57.36	63.55	86.25	91.95	90.57
13	10	84.19	88.43	89.00	98.70	98.32
14	63	92.57	97.89	98.23	99.43	98.85
15	19	64.65	81.57	83.04	90.94	98.24
16	5	81.85	92.98	85.42	96.13	98.04
**KAPPA**		0.754 ± 0.030	0.840 ± 0.025	0.935 ± 0.008	0.963 ± 0.005	0.966 ± 0.004
**OA (%)**		78.48 ± 2.58	86.04 ± 2.19	94.31 ± 0.65	96.76 ± 0.44	97.02 ± 0.30
**AA (%)**		64.74 ± 3.16	78.42 ± 2.87	89.01 ± 1.23	91.39 ± 2.09	92.34 ± 1.41

**Table 5:** Classification results of different models in Salinas.

No.	Training Samples	Res-2D-CNN	Res-3D-CNN	HybridSN	R-HybridSN	AD-HybridSN
1	20	59.97	97.63	99.90	99.99	99.81
2	37	99.48	99.82	100.00	99.97	100.00
3	20	60.01	92.35	99.48	99.54	99.98
4	14	98.27	98.87	98.59	99.13	99.17
5	27	94.80	96.85	99.08	98.92	99.50
6	39	99.89	99.98	99.59	99.93	100.00
7	36	97.21	98.80	99.96	99.70	99.97
8	113	83.53	87.19	99.13	98.44	99.70
9	62	99.26	99.55	99.97	99.99	100.00
10	33	84.95	93.58	98.70	97.84	98.96
11	11	90.00	91.44	98.34	99.04	99.22
12	19	97.15	99.26	99.68	99.89	99.92
13	9	95.74	97.74	97.21	94.93	95.59
14	11	94.84	98.29	97.58	93.51	97.49
15	72	72.28	78.62	98.45	96.84	99.57
16	18	91.12	87.12	99.85	99.45	99.00
**KAPPA**		0.862 ± 0.015	0.918 ± 0.010	0.992 ± 0.003	0.986 ± 0.003	0.995 ± 0.001
**OA (%)**		87.61 ± 1.38	92.68 ± 0.87	99.25 ± 0.31	98.74 ± 0.24	99.59 ± 0.10
**AA (%)**		88.66 ± 2.32	94.82 ± 0.98	99.09 ± 0.49	98.57 ± 0.42	99.24 ± 0.20

**Table 6:** Classification results of different models in the University of Pavia.

No.	Training Samples	Res-2D-CNN	Res-3D-CNN	HybridSN	R-HybridSN	AD-HybridSN
1	66	91.32	89.83	91.78	96.79	97.28
2	186	97.50	96.54	99.77	99.74	99.87
3	21	18.51	70.08	92.24	91.44	95.75
4	30	95.09	95.99	91.01	94.18	93.11
5	14	99.19	99.72	97.76	99.82	98.16
6	50	89.59	80.84	99.38	99.31	99.96
7	14	42.90	69.64	96.83	95.52	98.07
8	37	87.04	80.31	90.72	93.55	96.53
9	9	97.75	96.70	72.17	93.86	96.31
**KAPPA**		0.854 ± 0.012	0.870 ± 0.019	0.946 ± 0.013	0.969 ± 0.005	0.978 ± 0.004
**OA (%)**		89.025 ± 0.89	90.19 ± 1.42	95.94 ± 0.95	97.64 ± 0.38	98.32 ± 0.28
**AA (%)**		79.877 ± 2.75	86.63 ± 1.82	92.41 ± 2.14	96.02 ± 0.83	97.23 ± 0.61

**Table 7:** Classification results of R-HybridSN, D-HybridSN and AD-HybridSN.

		R-HybridSN	D-HybridSN	AD-HybridSN
**Indian Pines**	KAPPA	0.963 ± 0.005	0.958 ± 0.006	0.966 ± 0.004
	OA	96.76 ± 0.44	96.34 ± 0.50	97.02 ± 0.30
	AA	91.39 ± 2.09	91.58 ± 1.64	92.34 ± 1.41
Salinas	KAPPA	0.986 ± 0.003	0.993 ± 0.002	0.995 ± 0.001
	OA	98.74 ± 0.24	99.40 ± 0.20	99.59 ± 0.10
	AA	98.57 ± 0.42	99.33 ± 0.24	99.24 ± 0.20
University of Pavia	KAPPA	0.969 ± 0.005	0.972 ± 0.004	0.978 ± 0.004
	OA	97.64 ± 0.38	97.91 ± 0.27	98.32 ± 0.28
	AA	96.02 ± 0.83	96.74 ± 0.62	97.23 ± 0.61

**Table 8:** Classification results of the University of Pavia on an unbalanced training sample case.

	1%		0.8%		0.6%		0.4%	
	OA (%)	AA (%)	OA (%)	AA (%)	OA (%)	AA (%)	OA (%)	AA (%)
**HybridSN**	95.94	92.41	94.60	89.69	93.31	87.50	90.62	80.96
**R-HybridSN**	97.64	96.02	96.60	93.94	95.76	92.91	93.46	86.82
**AD-HybridSN**	98.32	97.23	97.57	95.61	97.24	95.40	96.13	92.15

**Table 9:** Classification results of the University of Pavia on an balanced training sample case.

	50		40		30		20	
	OA (%)	AA (%)	OA (%)	AA (%)	OA (%)	AA (%)	OA (%)	AA (%)
**HybridSN**	96.70	96.64	95.70	95.64	93.06	93.87	88.76	90.31
**R-HybridSN**	97.38	97.36	95.79	96.25	94.99	94.98	90.47	92.24
**AD-HybridSN**	98.32	98.31	97.14	97.36	96.33	96.70	93.98	94.59

## Supplementary Materials

The Indiana Pines, Salinas and University of Pavia datasets are available online at www.ehu.eus/ccwintco/index.php?title=Hyperspectral_Remote_Sensing_Scenes.
